# Pharmacological Treatment of Alzheimer’s Disease: Insights from *Drosophila melanogaster*

**DOI:** 10.3390/ijms21134621

**Published:** 2020-06-29

**Authors:** Xingyi Cheng, Chaochun Song, Yanjiao Du, Uma Gaur, Mingyao Yang

**Affiliations:** 1 Institute of Animal Genetics and Breeding, Sichuan Agricultural University, Chengdu 611130, China; chengxingyisicau@163.com (X.C.); songchaochun@outlook.com (C.S.); yanjiaodu177@hotmail.com (Y.D.); gaur.uma2906@gmail.com (U.G.); 2 Farm Animal Genetic Resources Exploration and Innovation Key Laboratory of Sichuan Province, Sichuan Agricultural University, Chengdu 611130, China

**Keywords:** Alzheimer’s disease, AD model, pharmacology, signaling pathways, *Drosophila*

## Abstract

Aging is an ineluctable law of life. During the process of aging, the occurrence of neurodegenerative disorders is prevalent in the elderly population and the predominant type of dementia is Alzheimer’s disease (AD). The clinical symptoms of AD include progressive memory loss and impairment of cognitive functions that interfere with daily life activities. The predominant neuropathological features in AD are extracellular β-amyloid (Aβ) plaque deposition and intracellular neurofibrillary tangles (NFTs) of hyperphosphorylated Tau. Because of its complex pathobiology, some tangible treatment can only ameliorate the symptoms, but not prevent the disease altogether. Numerous drugs during pre-clinical or clinical studies have shown no positive effect on the disease outcome. Therefore, understanding the basic pathophysiological mechanism of AD is imperative for the rational design of drugs that can be used to prevent this disease. *Drosophila* *melanogaster* has emerged as a highly efficient model system to explore the pathogenesis and treatment of AD. In this review we have summarized recent advancements in the pharmacological research on AD using *Drosophila* as a model species, discussed feasible treatment strategies and provided further reference for the mechanistic study and treatment of age-related AD.

## 1. Introduction

The fragile elderly population is the main socio-economic concern of society and it is expected that by 2050 there will be 1.6 billion people over the age of 65 worldwide [1]. The process of aging is often accompanied by the occurrence of various diseases which rarely affect younger individuals, such as cardiovascular disease, cancer, and neurodegeneration. Because of the complexity and the universality of neurodegeneration, studies involving its pathogenesis and treatment continue to invite a lot of attention from the scientific community [2]. Among all the neurodegenerative disorders, Alzheimer disease (AD) is the most common form of dementia, which causes cognitive deficits, memory loss, and behavioral alterations [3]. According to the World Alzheimer Report, about 50 million people worldwide were reported to have dementia in 2018. By 2050, owing to the increase in the elderly population, this number is expected to increase to 152 million, which is three times what it is now. The cost of global social dementia was estimated to be US $1 trillion in 2018, and this number will most likely increase to US$ 2 trillion by 2030 [4]. Unfortunately, the pathological mechanism of AD is still elusive, and no therapeutics are available for the prevention or mitigation of the disease progression.

## 2. Pathogenesis of Alzheimer’s Disease

**Figure 1:**
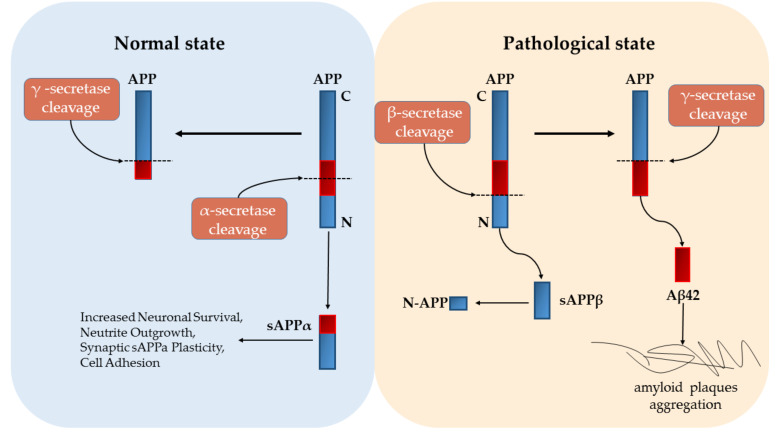
Amyloid precursor protein (APP) cleavage under normal and pathological states. Under normal state, APP is first cleaved by α-secretase to produce sAPP. sAPP can help the correct transduction of synaptic signals. Conversely, under pathological state, APP is sequentially cleaved by β-secretase and γ-secretase to produce Aβ42 fragment, which aggregates frequently to form plaques and eventually causes nerve cell death.

As one of the major neurodegenerative diseases, AD and its pathogenesis have been widely studied. Most of the recent studies on AD have been primarily focused on β-amyloid (Aβ) and Tau protein [5,6,7,8]. Meanwhile, biologically, AD is defined by the presence of a specific neuropathological profile, which is when extracellular deposition of β-amyloid forms intraneuronal neurofibrillary tangles and aggregated hyperphosphorylated tau protein [9,10]. Amyloid deposition, astrogliosis, tau protein hyperphosphorylation and accumulation, neuronal dystrophy, oxidative stress, decline in acetylcholine (ACh) levels, etc., constitute the main pathological hallmarks of AD [11,12,13,14,15]. By studying the intercellular molecular mechanism of AD, researchers found that Aβ, Amyloid precursor protein (APP) cleavage form and microtubule-binding protein tau are three important pathogenic molecules in AD pathology [16]. Three forms of secretase can cleave APP under different circumstances. Normally, APP is cleaved by α-secretase to produce sAPP (APPsβ) and a C83 carboxy terminal fragment, and the sAPP molecule establishes regular signaling with synapses to maintain synaptic plasticity [17]. However, according to the amyloid hypothesis, abnormal cleavage of APP by β-secretase (BACE1) and γ-secretase results in 42 amino acid long polypeptides (Aβ42 peptides), under pathological condition. Subsequently, the accumulation of Aβ42 in the plaque induces a pathological cascade and eventually leads to neurodegeneration (Figure 1) [18,19]. On the other hand, presence of NFTs is also a noticeable characteristic of AD, which is formed by hyperphosphorylated Tau. Multiple evidence has shown that aggregated, hyperphosphorylated forms of tau could also be a primary driver of AD and play a key role in promoting neuronal toxicity and neuronal loss [20,21,22]. Tau is a soluble protein that is naturally unfolded but, under ambient conditions, aggregates into oligomers and fibrils. Moreover, many kinases can mediate tau phosphorylation [23]. For example, inflammation in the brain can stimulate activation of mitogen-activated protein kinase (MAPK) which in turn activates the cyclin-dependent kinase-5 (CDK-5) leading to tau hyperphosphorylation [24].

A study by Oddo et al. suggested that clearance of Aβ42 reduced tau aggregation but increasing the expression of tau had no effect on Aβ42 pathology, suggesting that tau acts downstream of Aβ42 [25]. However, there is increasing information about the interaction between Aβ42 and tau [26]. Researchers should reconsider the relationship between Aβ42 and tau in order to discover tau-targeted therapeutics and to explore the mechanisms by which β-amyloid peptides elicit synaptotoxicity.

## 3. Application of *Drosophila* as Alzheimer’s Disease Model 

In order to facilitate an intensive understanding of the different pathways and pathogenesis involved in AD, many appropriate disease models have been developed and applied, including mouse, *Caenorhabditis elegans* and *Drosophila melanogaster* [27,28,29]. *Drosophila* is an excellent model for studying genetic and cell biology pathways in complex biological processes [30]. Owing to a complex brain, short lifespan and the relative ease in genetic manipulation, *Drosophila* can be a useful neurodegenerative invertebrate model [31]. 

**Figure 2:**
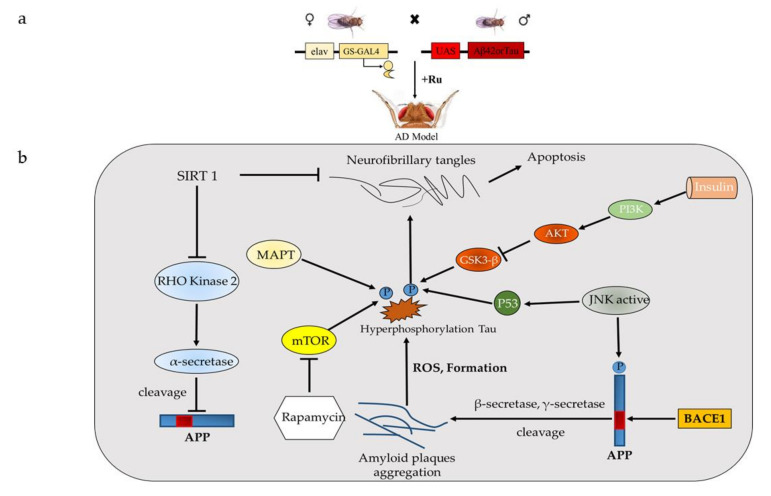
(**a**) Expression of Aβ42 peptide or tau protein in the *Drosophila* nervous system by UAS/GAL4 system (The yeast transcriptional activator Gal4 can be used to regulate gene expression in *Drosophila* by inserting the upstream activating sequence (UAS) to which it binds next to a gene of interest [47]). When females carrying a reporter gene with UAS (UAS-GFP) are mated with males carrying a GAL4 driver, progeny containing both elements of the system are produced. The presence of GAL4 in specific tissue will drive expression of the UAS reporter gene in a corresponding pattern. (**b**) Major signaling pathways involved in AD pathology of *Drosophila*.

Any vertebrate gene can be specifically expressed in specific tissues and cells of *Drosophila* using GAL4/UAS expression system [32], and nearly 70% of human pathogenic genes have homologous genes in *Drosophila* [33]. In order to study the associated mechanisms, the pathogenic gene responsible for AD can be inserted downstream of UAS to construct transgenic flies, which can overexpress the AD susceptibility gene at specific times and in specific tissues (Figure 2A) [34]. According to transgenes inserted, there are two main types of *Drosophila* AD model. The first and foremost type of *Drosophila* AD model is based on Aβ proteotoxicity. Although *Drosophila* contains homologous genes of human APP gene and an integral of the γ-secretase complex also has β-secretase, it cannot produce its own endogenous Aβ protein [35,36]. In that case, by over-expression of β-secretase-like protein, the cleavage of dAPP1 can be initiated to produce a fragment which is similar to human Aβ peptide and results in age-dependent behavioral deficits and neurodegeneration [35]. Along with inducing the endogenous production of Aβ peptide, the transgenic *Drosophila* producing Aβ42 peptide can also be used to study its toxicity and related neurodegeneration. Researchers constructed the transgenic *Drosophila* expressing human APP protein (HAPP), human β-secreted protein (hBACE) and *Drosophila* γ-secreted presenilin (dPsn), and showed an age-dependent neurodegenerative phenotype [37,38]. Interestingly, the toxic effect of detected Aβ42 in the fly was considerably higher in the APP-BACE1 transgenic flies compared to the Aβ42 transgenic flies, implying that the crucial points for Aβ42 proteotoxicity are the location and situation in which the peptide is generated and not the actual production level of Aβ42 peptide in the flies [39]. Simultaneously, tau plays a vital role in the formation of neurofibrillary tangles and neurotoxicity [40]. Aggregated and hyperphosphorylated forms of tau could be the primary driver of neurodegeneration in AD [41]. The second type of *Drosophila* AD model, the Tau-based transgenic *Drosophila* model, has been established, which can express abnormal human tau protein. This model displayed various symptoms of human degenerative diseases, including abnormal phosphorylation and aggregation of tau protein in neurons, and showed similar circadian and sleep deficits as seen in AD patients [42,43]. It has been suggested that aggregated Aβ42 stimulates tau phosphorylation and toxicity in AD pathogenesis, but the mechanisms are still elusive and many factors could cause tau phosphorylation [43]. Rising evidence supports the proposal that tau phosphorylation at Ser262 plays a critical role in Aβ42-induced tau toxicity in Aβ42 and tau co-expression *Drosophila* model [44]. Meanwhile, subsequent research showed that the loss of axonal mitochondria would phosphorylate tau at Ser262 which further induced Aβ42 toxicity in the pathogenesis of AD [45].

In the line with the above, most of the transgenic *Drosophila* AD models display a shortened lifespan [34]. So far, the most effective way to rescue AD has not been explored, but *Drosophila* models are often used to screen drugs with potential effects on lifespan shortening and AD phenotype [46]. In this review, we will focus on the main signaling pathways related to AD pathology and their corresponding pharmacological treatment using *Drosophila* models (Figure 2B). We hope to provide further inspiration for the development of new drug targets and new *Drosophila* AD models.

## 4. Pharmacological Treatment of Alzheimer’s Disease Using *Drosophila* as a Model

Numerous drugs have been tested to prevent and cure Alzheimer’s disease in an animal model [2,48,49]. The symptoms in most AD animal models are onset in adults; therefore, targeted drugs are considered to act directly on AD via ameliorating AD-related symptoms, rather than slowing down aging to delay the onset of AD [3,50]. We will discuss relevant drugs and their targeted signaling pathways in detail as follows.

### 4.1. IIS Signaling Pathway

The insulin/insulin-like growth factor 1 signaling pathway (IIS) is highly conserved and the most important signaling pathway known to regulate animal development and aging-related diseases. Evidence shows that insulin/insulin-like growth factor (IGF) signaling is important for neuron growth, synaptic maintenance, and neuroprotection [51], and insulin resistance is related to the tau hyperphosphorylation and increased level of Aβ42 [52], which is supposed to trigger AD. Diabetes is also a risk factor for AD and dementia, and insulin plays an important role in both conditions. Metformin has been used as an antidiabetic for many years. Early studies suggested that metformin reduces BACE1 transcription and Aβ production in neuronal cell lines, and found that this effect is insulin-dependent [52]. Recent study showed that metformin can promote new neuron growth in a series of in-vitro and in-vivo experiments [53]. Unfortunately, no evidence has shown benefits of metformin in the flies AD model, and some researchers have suggested that insulin signaling may just serve as a promising target for Alzheimer’s treatment in non-diabetes patients [54]. There is also some evidence that insulin treatment can meliorate cognition, but worsen AD pathology. That is why we need more irrefutable evidence to verify the connection between AD and insulin signaling. Recently, Huang et al. showed that Aβ causes accumulation of fly ILPs (Insulin-like peptides) and elevation of fly InR-S6K signaling, but knock down of ILPs, InR or the downstream components of S6K can effectively suppress Aβ toxicity. This indicates that utilizing S6K as a drug target to reduce insulin and insulin signaling can effectively reduce, instead of aggravating, the Aβ pathology. Although many upstream factors may alter Aβ toxicity, insulin signaling is the main downstream executor of Aβ toxicity. Therefore pharmacological intervention of the IIS pathway may serve as a promising target for the treatment of AD [55]. Along with S6K, there are some other important targets downstream of insulin signaling that influence AD pathology.

AKT plays important roles in neuronal survival, growth, polarity, synaptic plasticity and circuitry. Dysregulation of the AKT is the basis of many neurodevelopmental, neurocognitive, neuropsychiatric and neurodegenerative diseases [56]. Many studies have already proved that pharmacological inhibition of AKT activity can increase lifespan and healthspan in *Drosophila* [57]. Through genetic manipulations, Chen et al. created AKT RNAi and Aβ42 co-expressing *Drosophila* for pan-neuronal genes. Downregulation of AKT improved learning performance and increased the survival period in Aβ42 flies and, similarly, AKT inhibitor MK2206 also rescued the lifespan and learning deficit of Aβ42 flies [58]. A few other AD models also support this conclusion. After drug administration, nitazoxanide stimulated autophagy and promoted Aβ clearance by inhibiting AKT signaling in-vivo and in-vitro [59]. In contrast, decreased Aβ levels and Aβ deposition in brain were seen in salidroside fed Aβ-*Drosophila*, which protected neuron-cells from Aβ toxicity by upregulating AKT signaling [60]. Low dose Ionizing radiation suppressed the developmental defects and locomotive dysfunction, but had no effect on survival rates and longevity of Aβ42-expressing flies [61]. As far as the current results are concerned, the connection between AKT and AD becomes bewildering. AKT regulates neuronal survival and morphology and it is well established that with aging AKT activity increases. In that case, it is important to understand the role of abnormal AKT signaling in AD processing. It becomes imperative to explore whether the reduced activity of AKT can protect neuronal survival or increased activity is related to aging and promotes γ-secretase activity, leading to the cleavage of APP to and production of Aβ. In addition, it is necessary to underline whether increased Aβ activates more AKT to further increase γ-secretase activity and promotes APP processing [53]. We need more lines of evidence in order to explore appropriate drugs that target AKT.

Glycogen synthase kinase-3 (GSK-3) is a serine/threonine-specific protein kinase, also a major molecule involved downstream of insulin signaling. In the central nervous system (CNS), GSK-3β is the most abundant and is involved in diverse areas of physiology and pathology, including cellular metabolism and neurogenesis [62]. GSK-3β is the main kinase of tau protein, and its activation promotes tau phosphorylation and Aβ plaque deposition [63]. AD patients exhibit higher levels of circulating GSK-3β, therefore inhibition of GSK-3 activity has become a potential target for the treatment of various neurological diseases [64], and a variety of AD models have been developed to verify its effect. Lithium, a feasible GSK-3 inhibitor, is widely used in the treatment of psychiatric conditions. After administration, Lithium rescued Aβ toxicity caused by direct regulation of Aβ42 peptide levels but not mRNA levels [34]. Interestingly, this effect appears to be at least in part mediated by tau-independent mechanisms, and only at low dose prolonged the lifespan of mutant Aβ42 flies [65]. Flavonoid compounds Amentoflavone inhibited Aβ42 induced neurotoxicity in animal and cellular models through AMPK/GSK-3β-mediated pyroptosis suppression [66]. As mentioned before, BACE1 dysregulation results in AD pathogenesis, and GSK-3β regulates BACE1 transcription, thereby facilitating Aβ production in AD. AR-A014418 (ARA), a specific inhibitor of GSK-3β, reduced BACE1 promoter activity and gene expression in cell culture. In-vivo, ARA decreased BACE1 expression and markedly reduced β-secretase processing of APP and Aβ production, resulting in inhibition of neuritic plaque formation and amelioration of memory deficits [67]. Researchers proposed that by utilizing genetic manipulation to reduce GSK-3 in *Drosophila* larval neurons, AD induced synapse reduction can be recovered. In-vitro, GSK-3β inhibitor SB 415286 (SB) increased the density of synapses in hippocampal neurons in dose dependent manner [68]. Interestingly, a study in tau transgenic *Drosophila* model, after Salidroside treatment, showed improved longevity, locomotor activity, downregulated p-tau and fewer vacuoles in the mushroom body, but upregulated level of p-GSK-3β [49]. The catalytic activity of GSK-3β is regulated by phosphorylation at two different sites; phosphorylation at Ser9 site inactivates GSK-3β; however, phosphorylation at Tyr216 increases its catalytic activity [69]. This indicates that targets may have multiple regulatory sites and different sites may have completely different effects on the AD process. This is a big challenge for drug development. An exhaustive list of the drugs that target insulin signaling pathway to treat AD in *Drosophila* model is shown in Table 1.

### 4.2. mTOR Signaling Pathway

Mammalian target of rapamycin, mTOR, is a serine/threonine protein kinase, composed of two complex subtypes namely mTORC1 and mTORC2 [70]. Many studies have discussed the involvement of mTOR in life processes, including regulation of gene translation, development of neuronal stem cells, protein degradation, apoptosis and autophagy [71,72,73]. In CNS, the corresponding function of mTOR is regulated by many receptors or channels on the neuronal membrane [74]. 

In animal AD models, Aβ protein aggregation increases the expression of mTOR signaling, and conversely, reducing mTOR signaling can also bring down the expression of Aβ protein [75,76]. Rapamycin is a prevalent inhibitor of mTOR which effectively ameliorates neurodegeneration including improved cognitive deficit [77], increases Aβ clearance by reducing APP generation and downregulating β- and γ-secretase activity, and reduces Tau hyperphosphorylation by upregulating the levels of insulin-degrading enzyme [78,79]. Owing to the outstanding therapeutic benefits, rapamycin has been the center of disease research and drug development. For example, the long-term inhibitory effect of rapamycin on mTOR in AD mice, not only enhances learning and memory, but also regulates its behavior throughout life [80]. mTOR influences the onset and progression of neurodegenerative diseases by inhibiting autophagy [81,82] and autophagy can enhance neuronal survival by removing Aβ42 accumulation [83]. Hyperphosphorylated tau activates mTOR in AD flies, resulting in activation of cell cycle regulators and induction of neuronal death. A related study showed that reducing mTOR signaling expression or intake of rapamycin can reduce tau aggregate and improve related mobility impairments [84]. 

However, some reports suggested that knocking out of mTOR signaling impaired the synaptic plasticity, which can be reversed by up-regulation of mTOR and the loss of mTOR activity may also cause atrophy of AD neurons [85]. Salidroside treatment initiated mTOR activation in AD flies [60]. Considering the role of mTOR in memory formation and function in promoting cognition, it comes as no surprise that excessive activation as well as inactivation of mTOR might be the pathological mechanism behind the cognitive loss and AD progression. Therefore, it is necessary to keep mTOR at a precise balance point. In this case, treating AD with mTOR as the target, the timing of medication may be a crucial factor. Furthermore, the side effects of rapamycin and its analogue cannot be ignored, like reduced fertility and impaired locomotor activity [86]. Additionally, further research is needed to examine the beneficial effects of rapamycin in AD flies before deciding whether it can be a potential drug for clinical treatment of AD.

### 4.3. Sirtuin Pathway

Sirtuin, a cluster of conserved proteins encoded by the SIRT genes, is a type of protein that depends on NAD+ activation and has deacetylation activity [87]. The sirtuin family consists of seven members, including SIRT1, SIRT2, SIRT3, SIRT4, SIRT5, SIRT6, and SIRT7, which can regulate diverse physiological activities in aging cell [88]. Partly because activated sirtuins stimulate the activity of mitochondria, the powerhouses of the cell, and of mitochondrial proteins, they have the potential to regulate neurodegenerative diseases [89,90]. Here, we highlight and discuss the effect of SIRT1 and its activators on AD.

Owing to the multiple targets, SIRT1 plays an important role in neurodegenerative diseases [91]. During normal aging, SIRT1 is responsible for the maintenance of the nervous system and behavior, including regulation of synaptic plasticity and memory processes [92]. Activation of SIRT1 can down-regulate Aβ expression by reducing the formation of APP, and inhibiting RHO protein kinase II; to increase the activity of α-secretase and finally reduce the production of Aβ. Conversely, only inhibiting SIRT2 can have neuroprotective effects [89]. Resveratrol has long been used as a natural SIRT1 activator, and ameliorates AD in multiple ways [93,94]. In-vitro studies have shown that resveratrol treatment counteracts H_2_O_2_ induced damage, increases cell viability and delays the formation of Aβ42 oligomers and fibrils in cells [95]. Resveratrol exerts antioxidant effect against AD pathology, where it has been reported to disrupt Aβ aggregation via causing peptide fragmentation [96]. On the other hand, multiple in-vivo studies have indicated that resveratrol suppresses inflammation and inhibits toxicity of misfolded Aβ aggregation. It can rescue Aβ42-induced proliferation and activation of primary astrocytes and up-regulate SIRT1 expression to protect PC12 cells from Aβ_25-35_ neurotoxicity and apoptosis [97]. Simultaneously, a series of in-vitro studies showed that SIRT1 pharmacological activation can prevent amyloid deposition and neurodegeneration in AD [98]. For example, resveratrol protected neurotoxic p25 transgenic mice from CNS degeneration and cognitive decline, and ameliorated lifespan and cognitive impairment in mouse models [99]. Resveratrol has been shown to increase SIRT1 and AMPK in the brains of most transgenic mice [89]. 

Sirtuin is also highly conserved in *Drosophila melanogaster* and over-expression of sirtuin can extend the lifespan in *Drosophila*, yet some studies have shown contradicting results [100,101]. Bollinger et al. explored a resveratrol analogue called resveramorph 1, which has many functional group features similar to resveratrol. Results showed that resveramorph 1 protects synaptic function during acute oxidative stress at low dose and potentially contributes to the protection of synaptic transmission, which indicated that resveramorph 1 might confer its neuroprotective effects in a dose-dependent manner [102]. MPTP (1-Methyl-4-Phenyl-1,2,3,6-Tetrahydropyridine) is a neurotoxin which causes oxidative stress and inflammation in brain. The data showed that resveratrol increased lifespan in *Drosophila* and further ameliorated MPTP-triggered cell death, histological alterations, behavioral deficits and accumulation of nitric oxide and hydrogen peroxide levels in flies. A brain histology test showed that MPTP induced partial loss of neurons in the cerebral hemisphere rescued by resveratrol treatment, which indicated that the neuroprotective effect of resveratrol can improve oxidative stress and inflammation in the brain [103]. As mentioned above, AD and diabetes mellitus (DM) often coexist in patients, but mechanisms associated with DM and AD are bewildering. Nevertheless, some studies showed that Islet amyloid polypeptide (IAPP) can interact and co-deposit with Aβ and tau, thereby contributing to diabetes-associated dementia. This may be a clue to explain the relationship between AD and DM [104]. A recent study using rat models of diabetes with co-occurring AD, showed that resveratrol significantly increased the Sirt1 expression, inhibited the memory impairment, increased acetylcholinesterase, malondialdehyde, interleukin-1β and interleukin 6 levels, and decreased the levels of choline acetyltransferase (ChAT), superoxide dismutase (SOD), and glutathione [98]. All these reports point towards some kind of association between SIRT1 and AD pathology and further supported resveratrol as a potential drug to treat neurodegenerative disease. However, most investigations of resveratrol on AD were conducted in cell culture or animal AD models. The lack of information from human clinical studies is the major obstacle in proving the safety and efficacy of resveratrol for the treatment of human AD.

### 4.4. JNK Inhibitors

c-Jun N-terminal kinases (JNKs), first characterized as stress-activated members of the MAPK family [105], are evolutionarily conserved from fruit fly to human, and plays crucial roles in regulating a wide range of cellular activities including proliferation, differentiation and migration, and especially cell death, making it one of the most important targets in pharmacological screening strategies [106,107]. It seems that JNK is a key mediator of the Aβ-death pathway. Evidence proved that the early activation of JNK is always accompanied by the deposition of Aβ in the brain; JNK also co-localized with tau fibrillary tangle; and JNK activity is greater in the AD brains than control brains [108,109]. Study in a mouse AD model showed that electroacupuncture treatment significantly ameliorated the cognitive impairments and downregulated APP expression by inhibiting JNK activation [110]. Jang et al. explored an inhibitor called 1-phenyl-2-pyrimidyl-1H-benzimidazole derivative, which is a protein kinase inhibitor targeting JNK3. Further evidence showed that JNK3 is mainly expressed in the brain, indicating the potential of this compound for the treatment of AD [105]. In *Drosophila*, researchers expressed high levels of human Aβ42 polypeptide in the differentiating photoreceptor neurons and developed a transgenic model system in *Drosophila* eye [111]. A Chinese traditional medicinal prescription called KSOP1009 could strongly suppress Aβ42-induced eye degeneration and the locomotive dysfunctions via suppression of the hyperactivation of JNK activity and apoptosis [112]. A recent study found a positive feedback loop of Hippo and JNK signaling which regulates Aβ mediated neurodegeneration. Down-regulation of both signaling pathways can rescue Aβ mediated toxicity [113]. An early study also verified that Lunasin can downregulate JNK signaling dependent cell death in the developing retinal neurons of the *Drosophila* eye and reduce the mortality rate in AD flies [114]. On the other hand, gut microbiome dysbiosis seems to be related to AD pathology. It was reported that enterobacteria infection in *Drosophila* AD model declined lifespan, locomotor activity, and induced ROS stress, indicating that intestinal dysbiosis can remotely stimulate proinflammatory responses in the amyloid transgenic fly brain. The enteric infection promotes brain recruitment of hemocytes, which causes the activation of JNK and then the exacerbation of AD [115]. Recently, Chinese researchers developed a compound called GV-971, which targets gut microbiota and suppresses gut bacterial amino acids-shaped neuroinflammation to inhibit AD progression in mouse model, and also showed cognition improvement in a phase 3 clinical trial in China [116]. The advent of this drug makes up for the blank in the absence of new drugs to treat AD for more than a decade. However, we still need stronger evidence to show that gut microbiota may be an instructive therapeutic target for AD.

### 4.5. Natural Compounds and Antioxidants Target AD Pathology

Traditionally, natural plant products have been utilized for the treatment of various types of diseases, and the antioxidant activity they possess often reduces the toxicity induced by Aβ aggregation [117]. *Gardenia jasminoides* extract ameliorated memory deficits, and decreased the expression of inflammatory genes in AD *Drosophila* brain, but did not show any positive effects on lifespan extension and Aβ proteins reduction [118]. Treatment with nordihydroguaiaretic acid (NDGA), a phenolic lignin, rescued the shortened lifespan caused by AD in *Drosophila*. Furthermore, NDGA protected the hippocampal neurons against Aβ peptide induced toxicity by reduction of the oxidative stress [119]. It has been well established that the increased reactive oxygen species (ROS), lipid peroxidation (LPO), and enzymes such as SOD, are the biomarkers of AD pathology [120,121]. In this context, *Convolvulus pluricaulis* extract (aqueous) offsets tau induced early death and extends the lifespan and diminishes the level of tau protein in tauopathy *Drosophila*. Meanwhile, *C. pluricaulis* decreased the levels of ROS, LPO and oxidative damage of tau protein [122]. Similarly, luteolin reduced the level of LPO, SOD and inhibited Aβ42 plaque formation in Aβ42 expressing flies [50]. As a potent antioxidant, quercetin was able to effectively clear ROS and showed the beneficial effects in mouse AD model [123]. By modulating the expression of cell cycle related proteins, quercetin extended lifespan and rescued the climbing activity in AD flies. Owing to the fact that quercetin serve as Sirt1 agonist or acetylcholine-esterase (AChE) inhibitor and can target many signaling pathways, including BACE1, JNK signaling and GSK-3β in the brain, it can be safely stated that quercetin can be a useful compound for providing new insights into the treatment of neurodegeneration [124]. Polyphenols have been reported to suppress the activation of microglia and affect nuclear factor-kappaB (NF-κB) signaling to reduce inflammation [125]. For example, the exposure of AD flies to kaempferol showed delayed loss of climbing ability, memory, reduced the oxidative stress and acetylcholinesterase activity [126]. Curcumin, a bioactive polyphenolic compound, promoted amyloid fibril conversion by reducing the pre-fibrillar/oligomeric species of Aβ and down-regulating AChE gene expression [127], resulting in a reduced neurotoxicity in *Drosophila* and also exhibited an inhibitory effect on BACE1 activity [48]. Moreover, treatment with extract of *Arabidopsis thaliana* showed a lower pro-inflammatory and a higher anti-inflammatory effect. In-vivo, the extract significantly restored the locomotor activity of Aβ42 flies and exhibited neuroprotective effects [125]. Moreover, there are other natural compounds, such as flavonoids including myricetin, morin and rutin, which have been shown, in-vitro, to possess antiamyloidogenic and fibril destabilization activity, as well as being able to act as metal chelators and to suppress oxidative stress [12]. We have summarized the reports using natural compounds on *Drosophila* AD model in Table 2.

## 5. Conclusions

In past decades, the understanding of the pathological mechanisms of AD has improved, but there are still many basic questions that need to be answered. Here, we summarized recent advances in pharmacological treatment targeting major signaling pathways in *Drosophila* AD model, providing an exhaustive reference for the future drug development programs for the treatment of AD. Understanding the molecular mechanisms of disease through in-vivo animal model systems will help determine potential therapeutic targets. 

Due to the close genetic similarities to humans, *Drosophila* has been an important resource to investigate neurodegenerative diseases, including AD, Parkinson’s and Huntington’s disease. Compared to mouse, *Drosophila* needs less cost and space, and its shorter lifespan makes it possible to measure the efficacy of drugs within a reasonable time and allows implementation of a high throughput screen. On the other hand, due to the limitation of *Drosophila* AD models, it is necessary to develop more realistic AD models by new genome modification technologies, which can achieve better characterization of pathological mechanisms and quickly develop targeted drugs for AD. Unfortunately, no matter what kind of medicine, after dose-dependent and long-term administration, drug resistance or strong side effects often result. Therefore, in drug screening or development, a crucial point is to better understand the crosstalk between AD-related pathways, especially to understand which pathways can interact with each other and then to find relevant targets. Furthermore, the drug combinations need to be screened that can produce a synergistic effect, reduce the dosage of single drug or the cycle of medication, and produce the maximum effect with the lowest toxicity. In this regard, it is crucial to produce less toxic and safe drug candidates in the future. 

## Figures and Tables

**Table 1:** Overview of compounds targeting the insulin/insulin-like growth factor 1 signaling pathway (IIS) in *Drosophila* Alzheimer’s disease (AD) models.

Treatment	Mode of Action	Effects	References
MK2206	Inhibiting AKT activity	Improved Aβ42 induced early death and learning deficit	[58]
Salidroside	Upregulating AKT and GSK-3β activity	Decreased Aβ levels and Aβ deposition, protected neuron-cells	[49,60]
Ionizing radiation	Upregulating AKT activity	Suppressed developmental defects and locomotive dysfunction	[61]
Lithium	Inhibiting GSK-3 activity	Lifespan extension, rescued Aβ toxicity, reduced protein synthesis	[65]
SB 415286	Inhibiting GSK-3β activity	Promotes synapse formation	[68]

**Table 2:** Overview of natural compounds acting on *Drosophila* AD model.

Drug/Compound	Type of Molecule	Mode of Action	Effects	References
*Gardenia jasminoides*	Herbal extract	Decreased expression of inflammatory genes	Ameliorated memory deficits	[118]
*C. pluricaulis* extract	Herbal extract	Tau protein	Lifespan extension, decrease ROS and LPO level	[122]
NDGA	Phenolic lignan	Inhibitor of lipoxygenase, antioxidant	Lifespan extension, protected the hippocampal neurons	[119]
Luteolin	Polyphenols	Inhibition of AChE	Lifespan extension, rescue locomotive and prevention of Aβ42 plaque	[50]
Kaempferol	Polyphenols	Antioxidant	Rescue locomotive, improve memory and reduced AChE activity	[126]
Curcumin	Polyphenols	Inhibition BACE1 activity	Promotes amyloid fibril conversion, reduced neurotoxicity	[48,127]
*Arabidopsis thaliana*	Polyphenols	Activation Nrf2 pathway, antioxidant	Rescue locomotive and neuroprotective	[125]
Quercetin	Flavonoid	Cell cycle related proteins	Lifespan extension, rescue locomotive and restore Aβ induced perturbation	[124]

## Abbreviations

ACh	Acetylcholine
AChE	Acetylcholine-esterase
AD	Alzheimer’s disease
AKT	Protein kinase B
AMPK	AMP-regulated protein kinase
APP	Amyloid precursor protein
Aβ	Amyloid β
BACE1	β-secretase
CDK-5	Cyclin-dependent kinase-5
ChAT	Choline acetyltransferase
CNS	Central nervous system
DM	Diabetes mellitus
dPsn	*Drosophila* presenilin
GSK-3	Glycogen synthase kinase-3
HAPP	Human APP protein
hBACE	Human β-secreted protein
IGF	Insulin/Insulin-like growth factor
IIS	The insulin/IGF-1 signaling pathways
ILPs	Insulin-like peptides
InR	Insulin/IGF receptor
JNKs	c-Jun N-terminal kinases
LPO	Lipid peroxidation
MARK	Microtubule affinity regulating kinase
MAPK	Mitogen-activated protein kinase
MPTP	1-Methyl-4-Phenyl-1,2,3,6-Tetrahydropyridine
mTOR	Mammalian target of rapamycin
NDGA	Nordihydroguaiaretic acid
NFTs	Neurofibrillary tangles
NF-κB	Nuclear factor-kappaB
ROS	Reactive oxygen species
SOD	Superoxide dismutase
